# Sigmoid volvulus, initial manifestation of rectosigmoid invasive adenocarcinoma in a 52-year-old female: A case report

**DOI:** 10.1016/j.ijscr.2025.110872

**Published:** 2025-01-14

**Authors:** Siamak Mousazadeh, Saba Anvariazar, Sevil Ghasemi, Atabak Sedigh-Namin

**Affiliations:** a Department of Surgery, School of Medicine, Ardabil University of Medical Sciences, Ardabil, Iran; b Students Research Committee, School of Medicine, Ardabil University of Medical Sciences, Ardabil, Iran; c Iran University of Medical Sciences, Department of Surgery, School of Medicine, Tehran University of Medical Sciences, Tehran, Iran

**Keywords:** Sigmoid volvulus, Colon, Adenocarcinoma, Case report

## Abstract

**Introduction and importance:**

Sigmoid volvulus, an instead identified case of bowel obstruction, is defined as twisting a segment of sigmoid around its mesentery. Despite Western countries in which sigmoid volvulus affects the elderly, in volvulus belt countries, it is a disorder of predominantly men in their 4th decade of life.

**Case presentation:**

A 52-year-old woman presented with 4 days of abdominal distension and constipation. Physical exam showed tenderness and an empty rectum. Imaging confirmed sigmoid volvulus. Surgery revealed a large sigmoid tumor causing obstruction. The tumor was resected, and a colostomy was performed.

**Clinical discussion:**

Volvulus is a condition where the intestine twists, causing obstruction. It's often linked to diet, laxative use, and anatomical factors. Symptoms include abdominal pain and distension. In rare cases, it can occur with colon cancer.

**Conclusion:**

The tumor likely acted as a lead point, contributing to the development of volvulus in the long sigmoid colon.

## Highlights

• Sigmoid volvulus, often found in young men within "volvulus belt" countries, is rare in Western countries, where it primarily affects the elderly.
• The colonic adenocarcinoma in this case acted as a lead point, causing the volvulus, which is highly unusual.
• Radiologic imaging and surgery revealed an obstructive colonic tumor and significant bowel dilation.
• Histopathology confirmed the adenocarcinoma, underscoring the importance of cancer screening in cases of sigmoid volvulus with atypical presentations.

## 1 Introduction

This case report follows the SCARE criteria exclusively to ensure a structured and comprehensive presentation of the clinical scenario [1]. The term “volvulus” is derived from the Latin word “volvere,” meaning twist. Thus, sigmoid volvulus refers to the twisting of a segment of the sigmoid colon on its mesentery. In countries in the “volvulus belt,” sigmoid volvulus usually affects young men from the 4th decade onward with a male/female sex ratio of 4:1. In Western countries, sigmoid volvulus is preferentially observed among elderly males (age > 70) [2]. This case report aims to emphasize the rare occurrence of sigmoid volvulus as an initial manifestation of rectosigmoid adenocarcinoma. The challenges in writing this report included the limited existing literature on this uncommon presentation and the need to highlight its implications for clinical practice, particularly regarding diagnostic and therapeutic strategies.

## 2 Case report

A 52-year-old otherwise healthy female presented to the emergency center with complaints of gradually increasing abdominal distension and constipation for the past 4 days. During the history taking, it was revealed that she had been experiencing prolonged constipation and had no defecation or gas passing for four days. Physical examination showed generalized tenderness in the abdomen and an empty rectum.

**Fig. 1:**
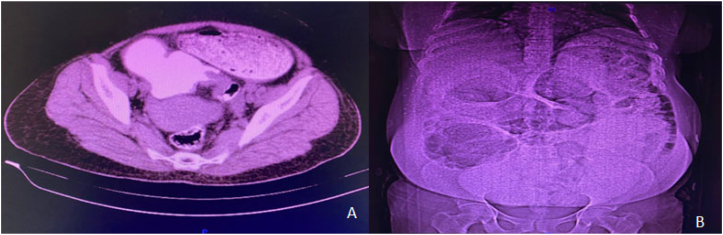
A. Computed tomography (CT) scan of the patient at presentation, demonstrating the transition zone of the volvulated sigmoid segment with associated bowel dilatation. B. Topographic CT scan of the patient showing the characteristic coffee bean sign, indicating a distended and volvulated sigmoid colon.

**Fig. 2:**
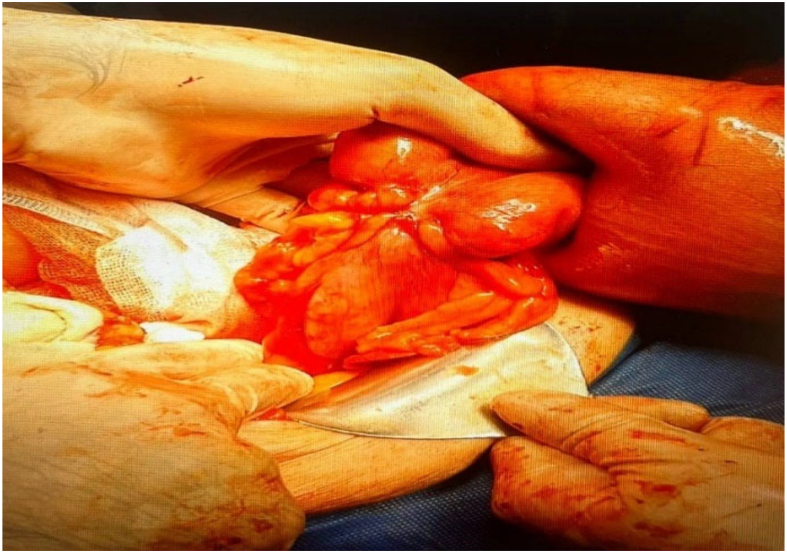
Shows intraoperative view of sigmoid colon cancer.

**Fig. 3:**
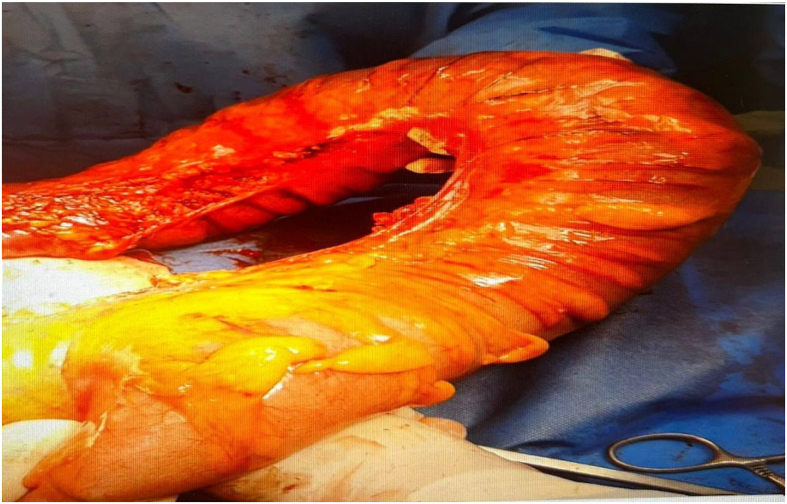
Shows intraoperative view of sigmoid colon cancer.

In the patient's abdominal CT scan and x-ray, dilation of a portion of the intestine and air-fluid levels were evident. Based on the CT findings (Fig. 1) and the patient's symptoms, a diagnosis of sigmoid volvulus was made. Thus, she scheduled for the surgery. After laparotomy, volvulus in the sigmoid colon and severe dilation of the proximal colon, along with obstruction at the distal end of the sigmoid colon caused by a large tumor, were observed (Fig. 2, Fig. 3). After performing devolvulation, the surgical team encountered a sizeable colonic tumor, which was resected with safe margins. Subsequently, a left hemicolectomy was performed, and a Hartmann's procedure was carried out, with a colostomy created for the patient. The mass was sent for histopathological study, and the patient recovered. The post-operative pathology report indicated the presence of a tumor in the sigmoid colon, identified as a well-differentiated adenocarcinoma. The tumor measured 5 × 3 cm and had invaded the pericolonic adipose tissue. Fortunately, there was no involvement of lymphatic vessels or nerves, and the surgical margins were free of cancerous cells. Additionally, none of the 11 examined lymph nodes showed metastatic involvement. The surgery lasted three hours, and since the patient showed no evidence of significant blood loss, no blood products were transfused. The patient's diet was initiated gradually after surgery following standard postoperative care protocols. On postoperative Day 1, the patient was allowed clear liquids to evaluate gastrointestinal tolerance. Upon confirming the absence of symptoms such as nausea, vomiting, or abdominal distension, the diet was advanced to full liquids on Day 2. From Day 3, a soft diet was introduced, which the patient tolerated well. The dietary plan was tailored based on the patient's clinical progress and overall condition. The patient was discharged in good general condition three days postoperatively.

The FOLFOX chemotherapy regimen was administered to the patient, consisting of Oxaliplatin, Leucovorin, and 5-Fluorouracil (5-FU). On the first day of each cycle, Oxaliplatin was given at a dose of 85 mg/m^2^ as an intravenous infusion over 2 h. This was followed by Leucovorin at a dose of 400 mg/m^2^, also administered intravenously over 2 h. Subsequently, 5-FU was administered in two phases: first as an intravenous bolus at a dose of 400 mg/m^2^, and then as a continuous infusion over 46 h at a dose of 2400 mg/m^2^ via an infusion pump. For the next two days (Days 2–3), the continuous infusion of 5-FU (2400 mg/m^2^) continued, with the patient receiving the drug through the infusion pump at home. From Day 4 to Day 14, a rest period was observed, during which no chemotherapy drugs were administered, and the patient was monitored for any side effects. This treatment regimen was followed for a total of 12 cycles, spanning approximately 6 months. Each cycle consisted of treatment on Day 1, followed by a rest period during the second week. The goal of this adjuvant chemotherapy was to reduce the risk of recurrence after surgery. Following the completion of treatment, the patient was closely monitored according to the standard follow-up protocol, tailored to individual patient needs and treatment regimens. During the first year after treatment, follow-up visits were scheduled every three months and included clinical examinations, blood tests (CEA levels), imaging studies (CT scans), and evaluation for close colostomy. From the second to third year, follow-up visits were extended to every six months, with continued clinical assessments, CEA monitoring, and CT scans conducted biannually. Throughout the follow-up period, no abnormal findings were observed, and the patient showed no evidence of disease recurrence.

## 3 Discussion

The term “volvulus” originates from the Latin word “volvere,” meaning twist, and was first described by Rokitansky in 1836 [2]. Colonic volvulus is the third leading cause of colonic obstruction globally, following colorectal cancer and complicated sigmoid diverticulitis [3]. In the United States and Western Europe, sigmoid volvulus is the most common form of colonic volvulus [4]. However, its incidence varies widely worldwide, being more prevalent in the “volvulus belt,” which includes regions such as Africa, South America, Russia, Eastern Europe, the Middle East, India, and Brazil, where it accounts for 13 % to 42 % of intestinal obstruction cases.

The etiology of colonic volvulus remains unclear, but it is thought to involve multifactorial causes such as chronic constipation, a high-fiber diet, frequent laxative use, and anatomical predispositions [5]. Clinical manifestations can range from asymptomatic cases to severe complications such as peritonitis secondary to colonic perforation.

The rarity of this case underscores the need for clinicians to maintain a high index of suspicion for malignancy in patients presenting with sigmoid volvulus, even in the absence of classical cancer symptoms. This approach ensures that underlying malignancies are not overlooked, which could delay appropriate treatment and worsen patient outcomes.

The co-existence of sigmoid volvulus and a tumor in the alimentary tract is exceedingly rare, with only two previous cases reported in the literature [6,7]. This case is particularly noteworthy due to the rare co-occurrence of sigmoid volvulus and rectosigmoid adenocarcinoma. It underscores the importance of considering underlying malignancies in patients presenting with acute bowel obstruction and highlights the need for heightened clinical suspicion.

Sigmoid volvulus typically affects elderly individuals or occurs in regions with high rates of chronic constipation, whereas colorectal cancer (CRC) is more commonly associated with symptoms such as weight loss, rectal bleeding, or changes in bowel habits. The simultaneous presence of these conditions in this patient highlights the importance of a broad differential diagnosis in acute bowel obstruction cases, especially involving volvulus.

In this report, CT imaging was instrumental in diagnosing the volvulated sigmoid segment and associated bowel dilatation. Although CT played a pivotal role, other diagnostic tools such as colonoscopy could have provided direct visualization of the tumor and further clarified the extent of obstruction. Similarly, contrast-enhanced imaging and magnetic resonance imaging (MRI) could offer valuable insights, particularly in cases involving complex bowel obstructions or associated tumors. The integration of these modalities, when feasible, can enhance preoperative planning and refine surgical decision-making.

The decision to perform Hartmann's procedure in this case was guided by the clinical presentation and intraoperative findings. This approach, which involves resection of the diseased segment with creation of a colostomy and closure of the rectal stump, is often chosen in emergency settings where the risk of anastomotic leakage is high. Given the patient's obstructive tumor and significant colonic dilation, primary anastomosis would have posed an increased risk of complications. Hartmann's procedure not only ensured immediate relief of the obstruction but also provided a safe option for managing the underlying malignancy. The success of this surgical intervention underscores its value in managing similar cases, particularly in high-risk or emergency scenarios [5,8,9].

Adjuvant chemotherapy, particularly the FOLFOX regimen, has been established as a cornerstone in the management of colorectal cancer (CRC) following curative surgery, especially in stage II and III disease. The FOLFOX regimen, which combines oxaliplatin, leucovorin, and 5-fluorouracil, has demonstrated significant improvements in disease-free survival (DFS) and overall survival (OS) in patients with resected colorectal cancer. Evidence from the MOSAIC trial and subsequent studies supports its role in reducing recurrence risk by targeting micrometastases [10,11].

In this case, despite the absence of lymph node involvement (pN0), the presence of pericolonic fat invasion (pT3) warranted adjuvant therapy to mitigate the risk of recurrence. The MOSAIC trial highlighted a 23 % relative reduction in mortality with the use of FOLFOX in similar patient populations, even among select stage II cases with high-risk features [10]. However, potential toxicities such as peripheral neuropathy and hematological suppression necessitate vigilant monitoring throughout treatment.

The patient in this case tolerated the 12 cycles of FOLFOX without significant complications, showcasing the importance of personalized supportive care during chemotherapy. This reinforces the role of adjuvant therapy in improving long-term outcomes and underscores the need for a multidisciplinary approach in managing colorectal cancer.

The favorable postoperative and follow-up outcomes in this patient emphasize the importance of timely surgical intervention and adjuvant chemotherapy in managing cases of bowel obstruction complicated by malignancy. This highlights the need for individualized treatment plans and close follow-up to ensure optimal long-term outcomes.

CRC represents a significant global health burden, ranking as the third most commonly diagnosed and the second most fatal cancer worldwide. It arises from the aberrant proliferation of glandular epithelial cells in the colon and is classified into sporadic, hereditary, and colitis-associated types. The progression of CRC involves stepwise genetic and epigenetic alterations in precursor lesions, such as adenomas and serrated polyps, which gradually develop dysplastic features and progress to adenocarcinoma [12] (Table 1).

**Table 1:** Scientific research and case presentations.

No.	Authors	Year	Age and sex	Clinical presentation	Imaging	Underlying pathology	Treatment
1	Lee et al.	2015	50/F	Lower abdominal pain and distension, bloody stool with a change in her stool caliber	CT: Wholly dilated bowel with gas and feces with whirl sign with rotation of the inferior mesenteric vessel and irregular wall thickening of the rectum, Colonoscopy: A circumscribed, ulcerofungating mass approximately 6 cm from the anal verge. The sigmoid colon was obstructed at a point approximately 25 cm from the anal verge. The mucosa was hyperemic and edematous with the pathognomonic spiral pattern of a volvulus.	Rectal adenocarcinoma	Unsuccessful endoscopic reduction Low anterior resection and protective ileostomy
2	Senejoa et al.	2022	63/M	Abdominal distension and absence of bowel movements and flatus	CT: a coffee bean image, as well as an area transition at the junction of the descending colon and the sigmoid with retractile changes suggestive of probable neoplastic involvement	Descending colon adenocarcinoma with carcinomatous seeding	Revolution of the sigmoid colon is performed with mesosigmoidoplasty, transverse colon decompression with Nelaton probe, rectal decompression with endorectal tube and colon loop colostomy transverse

## 4 Conclusion

The pathophysiology of this case highlights how the colonic tumor acted as a lead point for bowel obstruction, with increasing intraluminal pressure in the elongated sigmoid colon contributing to the development of volvulus.

## CRediT authorship contribution statement

Siamak Mousazadeh: Data collection, data analysis, and manuscript writing.
Saba Anvariazar: Study design, data interpretation, and manuscript review.
Sevil Ghasemi: Study concept and design, data interpretation, and manuscript review.
Atabak Sedigh-Namin: Study supervision, data interpretation, manuscript review, and corresponding author.

## Consent

Written informed consent was obtained from the patient for publication of this case report and accompanying images. A copy of the written consent is available for review by the Editor-in-Chief of this journal on request.

## Ethical approval

This study was reviewed and approved by the Research Ethics Committee of Ardabil University of Medical Sciences with the ethical code IR.ARUMS.REC.1403.096 on June 16, 2024.

## Guarantor

Atabak Sedigh-Namin accepts full responsibility for the work and the conduct of the study, had access to the data, and controlled the decision to publish.

## Research registration number

None.

## Funding

None.

## Declaration of competing interest

Authors have no conflict of interest to declare.
